# Zaleplon nanospanlastics loaded transdermal patches: formulation, optimization, ex-vivo permeation, and in-vivo studies

**DOI:** 10.3389/jpps.2025.15406

**Published:** 2025-11-05

**Authors:** Ahmed A. El-Shenawy, Reham A. Abd Elkarim, Reda A. Mahmoud, Abd El Hakim Ramadan, Ali H. Alamri, Hamdy Abdelkader, Mohamed S. Elafify, Essam A. Mahmoud, Mohammed S. Saddik, Mohamed S. Mohamed

**Affiliations:** ^1^ Department of Pharmaceutics and Pharmaceutical Technology, Faculty of Pharmacy, Al-Azhar University, Assiut, Egypt; ^2^ Al-Azhar Centre of Nano Sciences and Applications, Al-Azhar University, Assiut, Egypt; ^3^ Ministry of Health and Population, Assiut, Egypt; ^4^ Department of Pharmaceutics, Faculty of Pharmacy, Port Said University, Port Said, Egypt; ^5^ Department of Pharmaceutics, College of Pharmacy, King Khalid University, Abha, Saudi Arabia; ^6^ Department of Pharmaceutics and Pharmaceutical Technology, Faculty of Pharmacy, Menoufia University, Shebin El-Kom, Egypt; ^7^ Department of Clinical Pathology, Faculty of Veterinary Medicine, Zagazig University, Zagazig, Sharkia, Egypt; ^8^ Department of Pharmaceutics and Clinical Pharmacy, Faculty of Pharmacy, Sohag University, Sohag, Egypt; ^9^ College of Pharmacy, Al-Ayen Iraqi University, AUIQ, Nasiriyah, Iraq

**Keywords:** Zaleplon, spanlastics, factorial design, HPMC K100M, transdermal delivery

## Abstract

Zaleplon (ZLP) is a commonly used sedative-hypnotic drug that has low oral bioavailability because of its poor water solubility and extensive hepatic metabolism. This study aimed to encapsulate ZLP into spanlastic nanovesicles to enhance its bioavailability via transdermal delivery. Using Span 60 and Tween 80, ZLP-loaded spanlastics were fabricated using thin film hydration technique according to 3^2^ full factorial design. In the applied design, the influence of formulation variables on vesicle size, entrapment efficiency, and cumulative drug amount released over 24 h was investigated, leading to the identification of the optimal formulation. The optimized spanlastics were nanosized, spherical vesicles measuring 297.2 ± 8.17 nm, with an encapsulation efficiency of 65.75 ± 3.28% and a 24-hour drug release rate of 76.44 ± 5.66%. FT-IR studies revealed no significant chemical interactions between ZLP and the excipients used. HPMC K100M transdermal patches loaded with the optimized ZLP-spanlastics were formulated utilizing solvent casting technique. The patches were smooth and elastic with uniform drug content, ranging from 92.65 ± 2.54 to 96.12 ± 1.57%. The steady-state flux (Jss) of the spanlastic transdermal patch (ZLP-SP1) across rabbit skin was over 3.62 times higher than that of the control transdermal patch, indicating a significant enhancement in drug permeation. The investigated transdermal patches were stable under accelerated conditions with non-irritating properties. The *in-vivo* studies have shown that the pharmacokinetic parameters of ZLP oral suspension and ZLP-SP1 are significantly different, with the relative bioavailability of ZLP-SP1 being 2.681%. Therefore, these fabricated transdermal patches could be effectively used to treat insomnia.

## Introduction

Transdermal drug delivery systems (TDDSs) have the potential to deliver active pharmaceutical ingredients (APIs) through the skin for systemic effects [1]. Compared with conventional oral administration and hypodermic needles, TDDSs offer several properties such as reduced dosing frequency, improved patient compliance, ensuring uniform pharmacokinetic profiles, and reduced risk of side effects. In addition, they can be utilized to deliver wide variety of drugs. Moreover, the patient can terminate the medications at any time [2–5].

Scopolamine transdermal patches were the first commercially available patches for motion sickness management. Also, nicotine patches are available in the United States medical market. Fentanyl, nitroglycerine, lidocaine, and diclofenac epolamine topical patches are also available [6].

Among the various vesicular carriers developed to improve transdermal drug delivery, such as niosomes and liposomes, spanlastics are a novel type of elastic nanovesicle, consisting of nonionic surfactants and edge activators designed to enhance skin permeation [7, 8]. Spanlastics are made from a surfactant, mainly Span, and edge activators such as Tween 80 and ethanol, which affect the entrapment of the drug in the spanlastic vesicles and increasing the formulation stability by steric stabilization [9]. Spanlastics have several advantages over conventional vesicle systems, such as improved stability, biodegradability, elasticity, and deformability. Spanlastic-based drug delivery systems have been used to increase the delivery and penetration of various drugs into biological membranes such as Epigallocatechin gallate extracted from green tea, Fenoprofen calcium, Ascorbic acid, and Tacrolimus [10–13]. Spanlastics are amphiphilic molecules that could be utilized to encapsulate hydrophilic drugs in an inner hydrophilic core and hydrophobic drugs in an outer lipid layer [14].

Zaleplon (ZLP), N-[3-(3-cyanopyrazolo [1,5-a] pyrimidin-7-yl) phenyl]-N-ethylacetamide, is a hypnotic drug that acts by stimulating GABA_A_ receptor and is used for the short-term treatment of insomnia [15]. ZLP has a molecular weight of 305.54 g/mole. According to the biopharmaceutical classification system (BCS), ZLP belongs to a Class II drug (low aqueous solubility, 0.0403 mg/mL, and high intestinal permeability) [16]. Its bioavailability is only 30% due to extensive first-pass metabolism, and its elimination half-life is only 1 h [17]. Such properties make ZLP a suitable candidate for transdermal administration.

A previous study by Tirunagari et al., investigated the transdermal delivery of Zaleplon using matrix-based patches composed of HPMC and Eudragit polymers, incorporating permeation enhancers such as d-limonene to improve skin absorption. The current study explores a novel approach by utilizing nano spanlastic vesicles. These elastic nanocarriers are designed to improve drug solubility, stability, and skin permeation through their ultra-deformable structure [18].

This study aimed to develop ZLP-loaded spanlastics for incorporation into transdermal patches, with the objective of enhancing skin penetration and providing controlled release of ZLP. ZLP-loaded spanlastics were fabricated utilizing thin film hydration technique. A 3^2^ full factorial design was used to demonstrate the effect of different formulation variables on vesicle properties (encapsulation efficiency, vesicle size, and a 24 h *in-vitro* drug release). The optimized spanlastic formula was inserted into transdermal patches. The patches were formulated using different concentrations of film-forming polymer (i.e., hydroxypropyl methylcellulose) and solvent evaporation technology. The fabricated transdermal patches were estimated for their thickness, weight variation, drug content, elongation percent, and *ex-vivo* permeation study. The selected ZLP spanlastic transdermal patch was investigated for skin irritation and *in-vivo* assessments.

## Materials and methods

### Materials

Zaleplon was kindly provided by SIGMA for Pharmaceutical Industries, Egypt. Span 60, methanol (analytical and HPLC-grade), and Tween 80 were purchased from Sigma-Aldrich; St. Louis, MO, USA. Chloroform was provided by Fisher Scientific, UK. Hydroxypropyl methylcellulose (HPMC) K100M was supplied by Fluka Chemika, Switzerland. Polyethylene glycol 400 (PEG400) was purchased from Winlab, laboratory chemical; UK. Dialysis cellulose membrane (molecular weight cut off = 12,000 Da) was obtained from Sigma-Aldrich Co., USA. All other chemicals and reagents were of analytical grade and were used as it is.

### Methods

#### Fabrication of ZLP-Loaded spanlastics

ZLP-loaded spanlastics were prepared using the thin film hydration technique [19]. In a round-bottom flask, accurately weighed amounts of Span 60 (0.6, 1.2, or 1.8% *w/v*) and ZLP (10 mg) were dissolved in 10 mL of methanol: chloroform (1:3, *v/v*). After that, the organic mixture was vacuum evaporated at 60 °C utilizing a rotary evaporator (Heidolph vv2000, Germany) at a speed of 100 *rpm* to form a uniform film on the inner wall of the flask. Subsequently, the resulting dry film was hydrated with distilled water (10 mL) containing Tween 80 (0.6, 0.8, or 1% *w/v*) by rotating the flask in a 60 °C water bath at atmospheric pressure for 1 h and then kept overnight in refrigerator to allow proper swelling. The obtained dispersions were sonicated utilizing an ultrasonic processor, GE130, probe CV18, ZZKD machinery, China, for 10 min and then used for further investigation.

#### Optimization of ZLP-Loaded spanlastics

A 3^2^ full factorial design was utilized to optimize the fabricated ZLP-loaded spanlastics for desirable properties. The design model includes two factors at three levels, as shown in Table 1. Design Statgraphics centurion software, (Princeton, NJ, USA, version XVI) was used to generate nine formulations. The percentage of Span 60 (X_1_, % *w/v*) and the percentage of Tween 80 (X_2_, % *w/v*) were selected as independent factors to investigate their effects on the dependent responses: the entrapment efficiency (EE %) (Y_1_, %), vesicle size (ViS) (Y_2_, nm), and the cumulative percentage of ZLP released over 24 h (Q24h, h) (Y_3_, %) [20]. The results were examined statistically using ANOVA to indicate the impact of the independent factors on the responses at *P* < 0.05.

**TABLE 1:** The utilized 3^2^ Factorial-design.

Independent factors	Levels		
	High (+1)	Medium (0)	Low (−1)
X_1_; Span 60 (% *w/v*)	1.8	1.2	0.6
X_2_; Tween 80 (% *w/v*)	1	0.8	0.6

Dependent responses	Desirability constraints		
Y_1_; EE (%)	Maximization		
Y_2_; ViS (nm)	Minimization		
Y_3_; Cumulative % ZLP released over 24 h (%)	Maximization		

EE; entrapment efficiency, ViS; vesicle size.

#### Characterization of ZLP-Loaded spanlastics

##### Evaluation of ViS, polydispersity index (PDI), and zeta potential

The ViS and PDI of the fabricated ZLP-loaded spanlastic dispersions were evaluated by Zetasizer, Nano ZS, Malvern, UK. PDI is determined because it is a measure of the particle size uniformity of the prepared spanlastic formulation. A PDI value of less than 0.5 indicates a narrow particle size distribution [21]. The investigated samples were diluted with distilled water and assessed at a scattering angle of 90°. The same equipment was used to measure the zeta potential of the optimized formulation. The value of the zeta potential gives an idea about the potential stability of the colloidal system of nano sized spanlastic formulations. If all the particles in the colloidal spanlastic vesicles have a large negative or positive zeta value, then they will tend to repel each other and there will be no tendency for the particles in the suspension to aggregate together [22]. Three measurements were conducted at room temperature, and the mean value ±standard deviation (SD) was recorded.

##### Determination of EE %

The EE % of ZLP in the formulated spanlastics was characterized indirectly by measuring the amount of unentrapped ZLP. The samples of spanlastic formulations were centrifuged at 15,000 *rpm* for 1 h at 4 °C using a cooling centrifuge (Biofuge^®^ primo, Germany). The supernatant was separated and the samples were quantified for ZLP using high performance liquid chromatographic system (HPLC, Surveyor Plus, Thermo Fisher Scientific, USA) with the following conditions; RB-C18 column (4.6 mm × 150 mm, 5 μm), a mobile phase composed of acetonitrile, methanol, and 0.02 M potassium dihydrogen phosphate in a 25:15:60 (v/v) ratio, and detection at 240 nm [23]. EE % was assessed utilizing Equation 1.

$$\text{EE }\left(\%\right)=\frac{\text{Total}\;\text{ZLP}-\text{Free}\;\text{ZLP}\;\text{in}\;\text{supernatant}}{\text{Total}\;\text{ZLP}}\times 100$$ (1)

##### *In-vitro* ZLP release studies

The *in-vitro* ZLP release studies were conducted utilizing the dialysis bag diffusion method [24]. The dialysis membranes were soaked for 12 h in the distilled water before the study. An appropriate volume of the prepared ZLP-loaded spanlastic nanovesicles, equivalent to 10 mg ZLP was transferred into a dialysis bag, which was then securely sealed at both ends with cotton threads. The dialysis bag was immersed in a glass beaker filled with 100 mL of phosphate buffer solution (PBS), pH5.5 containing 20% isopropyl alcohol (IPA). The beaker was putted in a thermostatically controlled water bath shaker (Kotterman Labortechnik; GmBh, Germany). The rotation was adjusted at 50 *rpm* and the temperature was maintained at 37 ± 0.5 °C. Samples (2 mL) were withdrawn at the specified time intervals of 0, 1, 2, 3, 4, 5, 6, 8, 12, 18, and 24 h. The volume of each withdrawn sample was replaced by 2 mL of fresh and heated medium (PBS and IPA) to preserve a constant volume and ensure sink condition. The obtained samples were then quantified for ZLP using HPLC method. The release pattern of ZLP from the ZLP suspension was compared to that from the spanlastic formulations containing ZLP in the same manner.

##### Release-data analysis

For the determination of the appropriate kinetic model and the mechanism of the *in-vitro* release of ZLP from the fabricated spanlastic nanovesicles, various mathematical kinetic models, namely Zero-order, First-order, Higuchi-diffusion, and Korsmeyer-Pappas equations were utilized and the coefficient, R^2^ with the highest value was referred to the order of ZLP release [25].

##### Transmission electron microscope (TEM) imaging

Morphology of the optimized ZLP-loaded spanlastics nanovesicles formulation was examined utilizing JEM-100 CX 11, Electron microscope, Jeol, Tokyo, Japan. An optimum volume of the diluted formulation dispersion was applied to a carbon-coated copper grid and allowed to air drying at 25 °C. The sample was then examined and photographed by TEM at 80 kV.

##### Fourier-transform infrared (FT-IR) spectroscopy

The FT-IR spectra of the ZLP, span 60, Tween 80, and the optimized ZLP-loaded nanospanlastic formula were obtained utilizing Nicolet 6700, USA spectrophotometer. All samples were mixed with dry potassium bromide (KBr) and compressed in a hydraulic press before scanning between 4,000 and 400 cm^−1^.

#### Fabrication of ZLP-Loaded spanlastic transdermal patches (ZLP-SP)

Solvent casting method was used to fabricate ZLP spanlastic transdermal matrix-type systems [26]. Different concentrations of HPMC K100M aqueous solution (1, 1.5, 2% *w/v*) were used to fabricate ZLP-SP impregnated patches, and PEG400 was added as a plasticizer in a concentration (10% of the polymer weight). The optimized spanlastic formulation (equivalent to 100 mg of ZLP) was added to the aqueous polymer solutions. Afterwards, the magnetic stirrer was utilized for stirring of the mixture to attain homogeneity and sonicated for sufficient time to break air bubbles. Then, the obtained mixture was transferred into a Petri dish (diameter equal to 10 cm) and dried at room temperature. The dried patches were taken out, divided into strips of a certain size (4 cm^2^), in which the ZLP content was equivalent to 5 mg, and packed with aluminum foil. The fabricated patches were stored in a desiccator under ambient conditions until further evaluation. Table 2 shows the composition of different ZLP-SP transdermal patches.

**TABLE 2:** Composition of ZLP spanlastic-loaded transdermal patches.

Formula code	ZLP-spanlastics equivalent to	HPMC K100M (% *w/v*)	PEG400 (% *w/v*)
ZLP-SP1	100 mg of ZLP	1	0.1
ZLP-SP2	100 mg of ZLP	1.5	0.15
ZLP-SP3	100 mg of ZLP	2	0.2

#### Evaluation of ZLP-SP formulations

##### Physical appearance, thickness, pH, and weight uniformity

All the fabricated ZLP-SP formulations were visually inspected for *color, clarity, smoothness, and flexibility*. The *thickness* of the investigated ZLP-SP was measured using a digital caliber (Mitutoyo, Co., Sakado, Japan) at three different points. The obtained data was expressed as the mean values ±SD (n = 3). For estimation of the ZLP-SP *pH*, the investigated patches were soaked in distilled water (2 mL) and saved for 1 h in a beaker. The pH meter electrode (JENWAY- 3310, UK) was brought near the surface of the investigated ZLP-SP and pH readings were taken after allowing an equilibration period of 60 s. The fabricated ZLP-SP were subjected to *weight uniformity* test by weighing the investigated ZLP-SP (n = 3) on a digital sensitive electric balance (Radwag, Poland). The obtained data were recorded as the mean weight ±SD.

##### Drug content uniformity

The uniformity of ZLP distribution across the fabricated ZLP-SP formulations was evaluated by sampling sections of the patches from different locations, each measuring 4 cm^2^. The investigated ZLP-SP formula pieces were placed in a 25 mL volumetric flask and dissolved in isopropyl alcohol (10 mL) to disrupt the spanlastic vesicles and subsequently diluted with PBS, pH5.5 [9]. The obtained samples were filtered using filter paper, and the solutions were then quantified for ZLP using HPLC method. Three measurements were performed and the mean ± SD was listed.

##### Elongation percent (E %)

To evaluate the stretching ability of the fabricated ZLP-SP, the E % was estimated by observing the length just before the break point [27]. Square film specimens (5 cm × 5 cm) were prepared, and one end of the sample was secured to a fixed clamp, while the other end was attached to a string passed over a low-friction pulley. Incremental weights were gradually added to the free end until the patch broke. The E % was evaluated via Equation 2.

$$E\;\%=\frac{\left(\text{ZLP}-\text{SP}\;\text{final}\;\text{length}\right)-\left(\text{ZLP}-\text{SP}\;\text{initial}\;\text{length}\right)}{\text{ZLP}-\text{SP}\;\text{initial}\;\text{length}}\times 100$$ (2)

The experiment was performed on three ZLP-SP and the results were expressed as the mean value ±SD.

##### Moisture content investigation

The fabricated ZLP-SP formulations were accurately weighed and kept in a desiccator containing calcium chloride for 3 days at room temperature. Then the ZLP-SP under investigation were reweighed, and the % moisture content was calculated via Equation 3 [28].

$$\text{Moisture content }\left(\%\right)=\frac{\left(\text{ZLP}-\text{SP}\;\text{initial}\;\text{weight}\right)-\left(\text{ZLP}-\text{SP}\;\text{final}\;\text{weight}\right)}{\text{ZLP}-\text{SP}\;\text{final}\;\text{weight}}\times 100$$ (3)

##### Moisture uptake percentage study

Moisture uptake of the fabricated ZLP-SP formulations was investigated utilizing a climate chamber. The ZLP-SP formulations were stored in the chamber at 25 ± 1 °C and 75 ± 3% humidity for 72 h. The initial weights of the stored ZLP-SP formulations were recorded, and after 72 h they were reweighed again. Moisture uptake percentage was estimated based on the stored ZLP-SP formulations weight increase and recorded utilizing Equation 4 [29].

$$\text{Moisture uptake }\left(\%\right)=\frac{\left(\text{ZLP}-\text{SP}\;\text{final}\;\text{weight}\right)-\left(\text{ZLP}-\text{SP}\;\text{initial}\;\text{weight}\right)}{\text{ZLP}-\text{SP}\;\text{initial}\;\text{weight}}\times 100$$ (4)

#### *Ex-vivo* animal skin permeation studies

##### Preparation for rabbit skin

The *ex-vivo* permeation studies procedures were approved by the Ethical Committee of Al Azhar University, Faculty of Pharmacy; Assiut, Egypt (AZ-AS/PH-REC/46/2024). The rabbit hairless abdominal skin was supplied immediately after the sacrificing of the rabbit in a local poultry slaughterhouse. The epidermis was obtained by soaking the hairless rabbit skin in distilled water at 70 °C for 30 s. The subdermal skin layer was removed using forceps and the adhering fats were removed using a cotton piece impregnated with isopropyl alcohol. Then, the skin was washed three times with distilled water and kept in saline solution for 30 min before the beginning of the experiments.

##### *Ex-vivo* permeation studies utilizing Franz diffusion cell

The Franz cell apparatus (locally fabricated) was utilized to investigate the *ex-vivo* permeation pattern of ZLP from the fabricated transdermal patches. The effective diffusion rabbit skin surface area was 5.856 cm^2^, and the volume of the receiving chamber was 30 mL of PBS, pH5.5 containing 20% isopropanol. The rabbit skin dermal side was placed facing the receptor compartment. The investigated transdermal patches (loaded with either the ZLP-spanlastic dispersion or ZLP dispersion) with a surface area of 4 cm^2^ equivalent to 5 mg of ZLP were placed on the surface of the rabbit skin in the direction of the donor compartment. The receptor medium was stirred by magnetic bar at temperature 32 ± 0.5 °C (similar to skin temperature) and speed of 50 *rpm* [30]. To prevent the diffusion medium evaporation, the donor compartment was covered with aluminum foil. At specified time intervals (1, 2, 3, 4, 5, 6, 8, 12, 18, and 24 h); samples (1 mL) were withdrawn from the receptor compartment and subrogated by an equal volume of the same medium. Samples were quantified for ZLP content utilizing HPLC method. The cumulative amount of ZLP that permeated through the rabbit skin was plotted vs. time (t). The steady-state flux (Jss, µg/cm^2^h) was determined from the slope of the linear portion of the cumulative amount permeated per unit area vs. time. The apparent permeability (P) was determined utilizing Equation 5 [26].

$$P=\frac{J_{SS}}{C_{i}}$$ (5)

Where, C_i_ is the initial ZLP concentration in the donor compartment of the Franz diffusion cell.

Enhancement ratio (ER) (Equation 6) was utilized to compare the ZLP flux from ZLP-SP formulations and pure ZLP-loaded transdermal patches.

$$\text{ER}=\frac{Jss\;\left(ZLP-SP\;formulation\right)}{Jss\;\left(Pure\;ZLP\;transdermal\;Patches\right)}$$ (6)

#### Skin irritation testing

Fifteen healthy adult Albino New Zealand rabbits were utilized for skin irritation studies. The animals were stayed in polypropylene coops and given a standard laboratory diet and water at 25 ± 1 °C with a 12 h light/12 h dark cycle. The procedures were approved by the ethical approval number: AZ-AS/PH-REC/46/2024. The animals were divided equally into three groups: group (A) (negative control group), group (B) (positive control group) and group (C) (treatment group). Before the study, the back skin of each group of animals with an area of about 3 cm × 3 cm was shaved with an electric shaver. The negative control group received a placebo transdermal patch, the positive control group received 1% *v/v* formaldehyde in water as a standard skin irritant, and the treatment group received the selected ZLP-SP (ZLP-SP1) formulation. The transdermal patch was covered with gauze and wrapped with adhesive tape. The applied transdermal patch remained in contact with the skin for 12 h, after which it was removed. The animals were given new transdermal patches daily for 7 successive days. Skin reactions were defined according to Organization for Economic Cooperation and Development (OECD) guidelines [31]. The erythema was scored on a scale of 0–4 as listed in Table 3 [32].

**TABLE 3:** Erythema scores.

Score	0	1	2	3	4
Erythema degree	No erythema	Very slight	Well-defined	Moderate to severe	Severe
Erythema color	-	Light pink	Dark pink	Light red	Dark red

#### *In-vivo* assessment

Three groups (A, B, and C) each containing 5 healthy adult Albino New Zealand rabbits weighing 1.8–2 kg were used. They were kept in an animal house at room temperature with free access to food and water. The negative control group, group (A) received no medication, group (B) received the selected ZLP-SP formulation (ZLP-SP1), and group (C) orally received ZLP suspension (2.5 mg/kg) through gastric tube [33]. For group (B), hair was removed from the dorsal region using electric clippers, followed by the application of the investigated ZLP-SP formulation. For the group (C), the animals were fasted for 12 h prior to the experiment beginning. Blood samples were then collected from the tail vein at predetermined time intervals over a 24 h period (0, 1, 2, 4, 6, 8, 12, 16, 20, and 24 h). The blood samples were centrifuged for 10 minutes at 4,000 *rpm* to separate the plasma. For ZLP extraction, 100 µL of 2M sodium hydroxide solution and 5 mL ethyl acetate were added to 1 mL of plasma sample.

The mixture was then vortexed for 5 min and then centrifuged for 10 minutes at 3000 *rpm*. The organic layer was separated and transferred to a new tube and evaporated to dryness under nitrogen flow. The residue was dissolved in 100 μL mobile phase (acetonitrile: water = 70:30, *v/v*). An aliquot of 20 μL was injected into the HPLC system for estimation of ZLP concentrations [34].

##### Pharmacokinetic parameters

The pharmacokinetic parameters of ZLP were evaluated using the plasma concentration-time curves; maximum plasma concentration (C_max_), time to reach maximum plasma concentration (t_max_), elimination rate constant (K_el_), and half-life (t_0.5_). The area under the plasma concentration-time curve from zero to the end of sampling time (AUC_0-24_) and from zero to infinity ( ${\text{AUC}}_{0-\infty }$ ) was calculated using the trapezoidal method. All the investigated parameters were presented as the mean values ±SD. The relative bioavailability of the transdermal patch to the oral suspension was computed using Equation 7 [35]:

$$\text{Relative bioavailability }\%=\frac{{AUC}_{0-24\;}\left(transdemal\;patch\right)}{{AUC}_{\;0-24}\left(oral\;suspension\right)}\times \frac{Dose\;of\;ZLP\;suspension}{Dose\;of\;ZLP\;transdermal\;patch}\;\%$$ (7)

#### Stability studies

Stability studies for the selected ZLP-SP formula (ZLP-SP1) were conducted for a period of 3 months. The stability investigation was carried out at 40 ± 0.5 °C and 75 ± 5% relative humidity (RH) [36]. The physicochemical parameters of the tested ZLP-SP formulations were evaluated at the end of the study time (3 months). Triplicate manner measurements were tabulated as the mean ± SD (n = 3).

#### Statistical analysis

Vis, zeta potential, drug content, E %, EE %, and cumulative % ZLP amount released were presented as means ± SD and treated statistically using one-way analysis of variance (ANOVA). The statistical significance of differences between the pharmacokinetic parameters of the selected transdermal patches and ZLP oral suspension was determined using one-way analysis of variance (ANOVA). The area under the curve (AUC) assessment was conducted using trapezoidal rule with baseline correction. *P* < 0.05 was considered statistically significant.

## Results and discussion

### Development of ZLP-Loaded spanlastic dispersions

ZLP-loaded spanlastic nanovesicles were successfully fabricated by the thin film hydration method using Span 60 and Tween 80. Span 60 is responsible for the formation of unilamellar and/or multilamellar vesicles, while Tween 80 acts as an edge activator to promote the deformation properties of spanlastics [37, 38]. A 3^2^ full factorial design was used, and statistical analysis was performed to determine the effect of these two variables on the properties of the formulated spanlastics. The measured responses of the nine runs are displayed in Table 4.

**TABLE 4:** Experimental runs of independent factors, dependent responses, and PDI of ZLP-loaded spanlastics.

Run	Independent factors		Dependent responses			PDI
	Span 60 (%*w/v*)	Tween 80 (%*w/v*)	EE (%)	VS (nm)	% Released at 24 h (%)	
F1	1.2 (0)	0.6 (−1)	61.09 ± 1.78	447.2 ± 9.80	68.77 ± 4.17	0.143 ± 0.030
F2	1.2 (0)	0.8 (0)	54.35 ± 2.11	318.3 ± 10.5	62.13 ± 5.02	0.440 ± 0.040
F3	0.6 (−1)	0.6 (−1)	43.78 ± 2.28	198.1 ± 5.10	80.89 ± 4.44	0.230 ± 0.060
F4	0.6 (−1)	0.8 (0)	40.51 ± 4.65	145.8 ± 6.60	77.02 ± 3.26	0.212 ± 0.005
F5	0.6 (−1)	1.0 (+1)	37.89 ± 3.32	103.2 ± 5.70	72.13 ± 4.47	0.175 ± 0.010
F6	1.8 (+1)	0.8 (0)	66.89 ± 2.79	504.8 ± 12.6	48.67 ± 2.69	0.487 ± 0.070
F7	1.2 (0)	1.0 (+1)	50.22 ± 3.47	245.8 ± 4.90	58.86 ± 3.07	0.315 ± 0.001
F8	1.8 (+1)	0.6 (−1)	71.54 ± 3.60	574.3 ± 11.3	52.17 ± 5.11	0.282 ± 0.002
F9	1.8 (+1)	1 (+1)	64.11 ± 5.35	469.5 ± 15.8	42.86 ± 3.78	0.411 ± 0.050

Values are represented as the means ± SD, EE; entrapment efficiency, VS; vesicle size, PDI; polydispersity index.

#### Effect of formulation variables on ViS

In the development of vesicular carriers for transdermal drug delivery systems, vesicle size plays a crucial role, as it directly affects the ability of vesicles to penetrate through the skin layers. Therefore, preparing nanovesicles with smaller vesicle size to ensure deeper penetration is an important goal of our current research.

The size range of the prepared ZLP-loaded spanlastic nanovesicles ranged between 103.2 ± 5.7 (F5) and 574.3 ± 11.3 (F8) nm, Table 4. The following equation for the vesicle size was obtained by the design software:

Vesicle size (nm) = 455.922 + 352.361×Span 60 - 978.167×Tween 80 - 12.4537×Span 60^2^ - 20.625×Span 60×Tween 80 + 417.917×Tween 80^2^.

**FIGURE 1:**
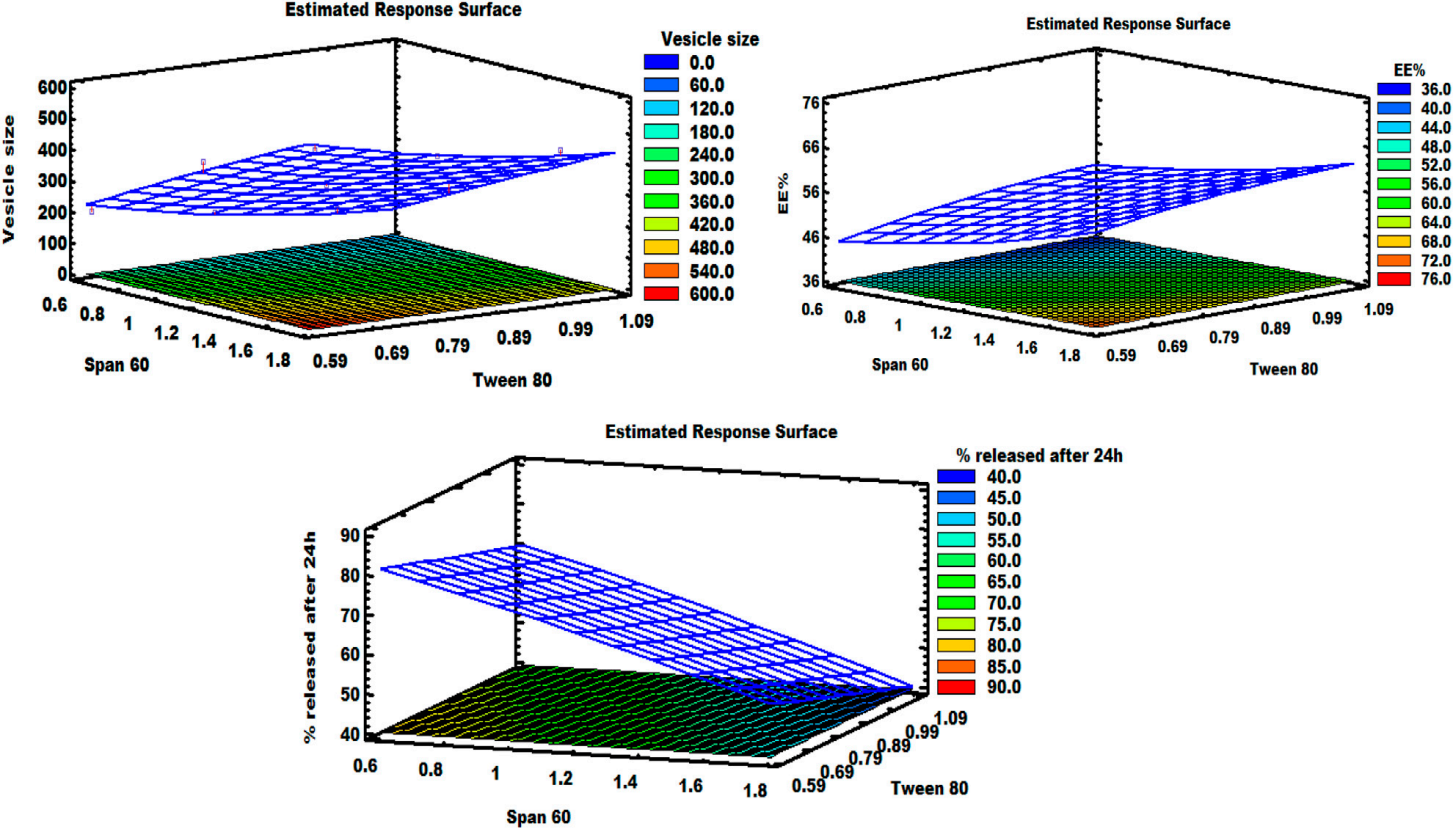
Response surface graph demonstrating the effect of independent factors on various responses.

**FIGURE 2:**
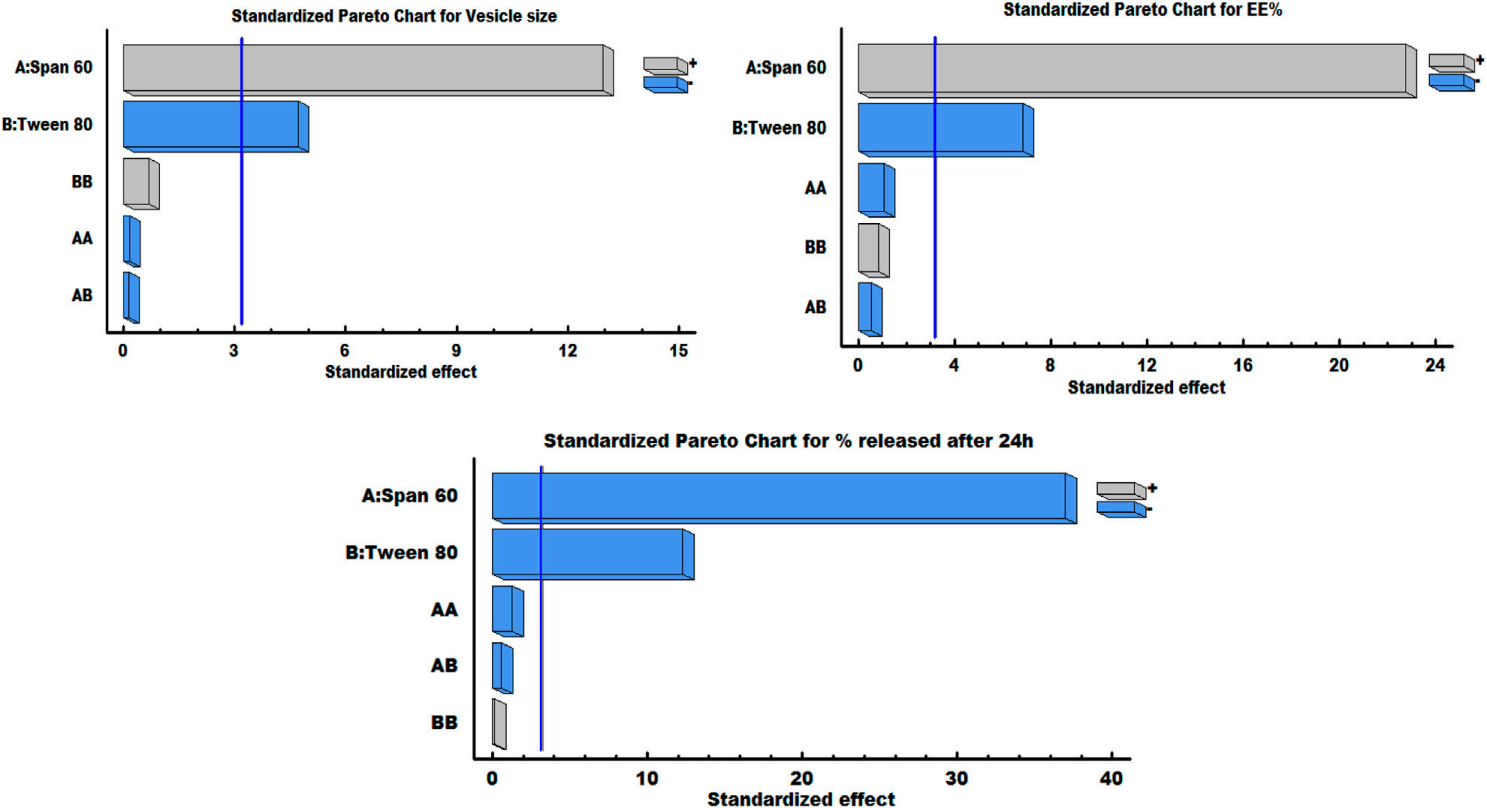
Pareto charts illustrating the significant impact of Span 60 and Tween 80 on various responses.

It can be noted that the Span 60 concentration has a synergistic effect on the vesicle size, while the Tween 80 concentration has an antagonistic effect on the same response. As shown in the response surface plot (Figure 1), increasing the concentration of Span 60 led to an increase in the size of spanlastic vesicles, whereas increasing Tween 80 concentrations resulted in a decrease in vesicle size. According to the Pareto diagram (Figure 2) and the ANOVA results (Table 5), two variables were found to have a significant impact on the vesicle size (*P* < 0.05). The smaller vesicle size observed with increasing Tween 80 concentration may be related to a decrease in surface free energy, which results in an inhibition of vesicle aggregation and thus a decrease in vesicle size. In addition, Tween 80 is small in size and has an unsaturated site, which maximizes the flexibility of its chain and facilitates its incorporation into nanovesicles to obtain a smaller size. In contrast, lower concentrations of Tween 80 may not cover the entire vesicle surface, which may cause vesicle coalescence to produce larger vesicles [39]. For Span 60; the increase in vesicle size can be attributed to the insertion of more alkyl chains into the hydrophobic part of the vesicle, resulting in an increase in vesicle size by increasing Span 60 concentrations [40]. Similar findings were reported in the formulation of Miconazole-loaded spanlastics [41].

**TABLE 5:** Analysis of variance for ViS, EE %, and % released at 24 h of ZLP-loaded spanlastics.

Source	ViS (nm)			EE %			% Released at 24 h		
	Sum of squares	F-ratio	P-value	Sum of squares	F-ratio	P-value	Sum of squares	F-ratio	P-value
A:Span 60	202,217	167.61	0.0010	1,076.29	517.90	0.0002	1,226.94	1,367.48	0.0000
B:Tween 80	26,813.5	22.23	0.0181	97.526	46.93	0.0064	135.565	151.09	0.0012
AA	40.2006	0.03	0.8668	2.42	1.16	0.3596	1.52542	1.70	0.2833
AB	24.5025	0.02	0.8957	0.5929	0.29	0.6303	0.297025	0.33	0.6054
BB	558.894	0.46	0.5449	1.46205	0.70	0.4632	0.0186889	0.02	0.8944

The PDI is utilized to investigate the particle size distribution behavior of the prepared formulations [42]. As shown in Table 4, the PDI values of the prepared ZLP-loaded spanlastic formulations ranged from 0.143 to 0.487, indicating a uniform distribution of dispersed vesicles.

#### Effect of formulation variables on EE %

In fact, the formulation of ZLP-loaded spanlastic nanovesicular dispersions with high EE % presents a significant challenge. ZLP was successfully entrapped in all the prepared spanlastic vesicles, ranging from 37.89 ± 3.32 to 71.54 ± 3.60%, Table 4.

The analysis of the data statistically showed significant effects (*P* < 0.05) of the surfactant and EA concentrations on EE % of spanlastic formulations, Table 5. The relationship between EE % and the two variables was explained by the polynomial equation given below:

EE % = 50.19 + 32.2222×Span 60 - 50.5083×Tween 80 - 3.05556×Span 60^2^ - 3.20833×Span 60×Tween 80 + 21.375×Tween 80^2^.

As shown in the equation, span 60 has a positive effect on EE %, while Tween 80 has a negative effect on the selected response. The Pareto plot (Figure 2) and ANOVA results (Table 5) confirmed that these effects were significant (*P* < 0.05). It was observed from the response surface graph (Figure 1) that as the Tween 80 concentration was increased, the EE % decreased; on the contrary, as the Span concentration was increased, the EE % continued to increase. These results indicate that Span 60 is important for better trapping of ZLP into vesicles [43]. These may be assigned to the lipophilicity and high phase transition temperature of Span 60, resulting in reduced fluidization of the vesicle membrane, thereby reducing the leakage of entrapped ZLP and thus increasing entrapment efficiency [44]. On the other hand, the lower EE % values obtained with increasing Tween 80 may be related to the higher HLB value of Tween 80, in addition to the unsaturation of its alkyl chain increasing its hydrophilicity, leading to increased fluidity of the vesicle membrane and increased permeability of ZLP from spanlastic vesicles [45]. These results are in line with those reported for Zolmitriptan spanlastics [46].

#### Effect of formulation variables on the Q24 h of ZLP spanlastics

**FIGURE 3:**
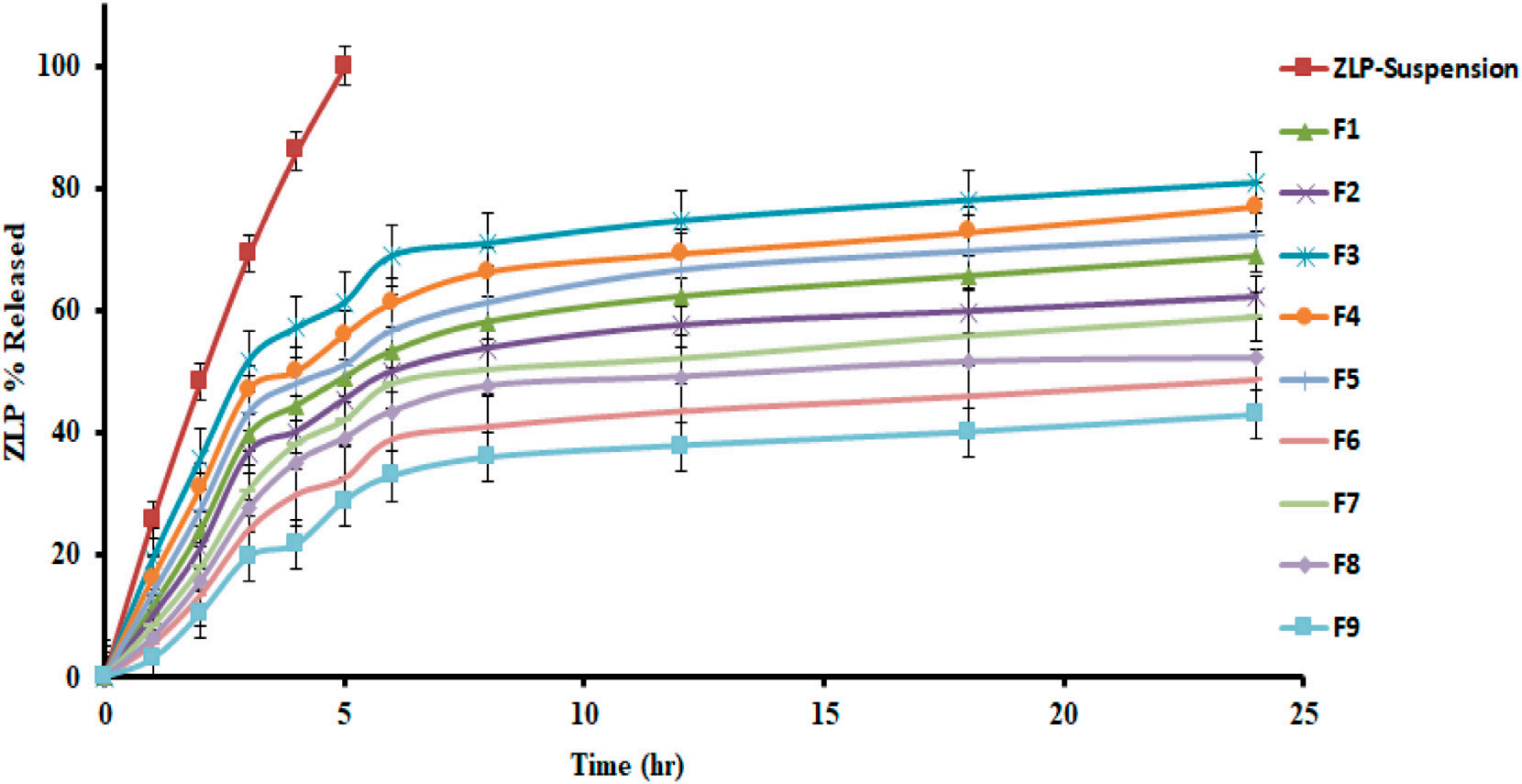
*In-vitro* release profiles of different ZLP-loaded spanlastic vesicular formulations compared to pure ZLP suspension.

*In-vitro* drug release of the studied spanlastic formulations is important to verify the consistency of the final product to obtain an ideal system with the desired release characteristics. Figure 3 shows the *in-vitro* release profiles of the prepared ZLP-loaded spanlastic formulations compared to the pure ZLP suspension. The results obtained indicate that ZLP-spanlastics had a slower release than drug suspension. The release of ZLP from spanlastic is biphasic, with a relatively rapid release lasting for 4 h, followed by a stable phase with a sustained release rate lasting for 24 h. The initial fast-release pattern may be attributed to the diffusion of unentrapped ZLP, which may be adsorbed on the surface of the spanlastic vesicles. The long-lasting sustained release pattern can be assigned to the diffusion ZLP through the spanlastic vesicles membrane bilayer and into the release media. Comparable results were observed in a previous study on Rutin spanlastics [47]. The effect of different independent factors on % ZLP released over 24 h from the prepared spanlastic vesicles dispersions is shown in Figure 1. The graph showed that the % ZLP released decreased with increasing Span 60 and Tween 80 concentrations. Statistical analysis manifested that these variables had a significant effect (*P* < 0.05) on the percentage of ZLP released from the prepared formulations, Table 5. The quadratic equation generated by the design was as follows:

% released over 24 h = 106.676–16.1944×Span 60 - 24.9083×Tween 80 - 2.42593×Span 60^2^ - 2.27083×Span 60×Tween 80 + 2.41667×Tween 80^2^.

It is worth noting that Span 60 and Tween 80 had a negative impact on the % ZLP released after 24 h, which can be seen from the Pareto chart (Figure 2).

The decrease in drug release upon increasing the amount of Tween 80 may be due to the loss of the vesicular structures and the formation of mixed micelles, which have poorer penetration properties compared to spanlastics [48]. Additionally, Span 60 is an HLB 4.7 hydrophobic surfactant with long chains that form stable vesicles in which the drug is more easily trapped instead of penetrating into the aqueous external phase, thereby delaying drug release [49]. The kinetic analysis illustrated that the release of ZLP from spanlastic formulations fitted best with Higuchi’s diffusion model, which has the highest R^2^ values. Further analysis of the release data using the Korsemeyer-Peppas equation indicated both Fickian and non-Fickian diffusion mechanisms based on the n (diffusion exponent) values, as shown in Table 6.

**TABLE 6:** Kinetic analysis of the fabricated ZLP-loaded spanlastics formulations.

Run	Zero-order		First-order		Diffusion		Korsmeyer	Fitted model
	R^2^	K_0_	R^2^	K_1_	R^2^	K_H_	n	
F1	0.79813	2.3863	0.8774	0.01928	0.93102	14.7922	0.51130	Diffusion
F2	0.78522	2.1695	0.8494	0.01603	0.92187	13.5355	0.52457	Diffusion
F3	0.75515	2.5938	0.8704	0.02680	0.90930	16.5975	0.40786	Diffusion
F4	0.78343	2.5496	0.8849	0.02384	0.92536	16.0035	0.44488	Diffusion
F5	0.79257	2.4677	0.8804	0.02126	0.92975	15.3831	0.47776	Diffusion
F6	0.80904	1.7904	0.8503	0.01119	0.93031	10.9407	0.63129	Diffusion
F7	0.79364	2.0910	0.8500	0.01465	0.92357	12.9307	0.56678	Diffusion
F8	0.77575	1.9043	0.8189	0.01249	0.91117	11.8864	0.60263	Diffusion
F9	0.82579	1.6417	0.8585	0.00964	0.93518	9.87992	0.74672	Diffusion

**FIGURE 4:**
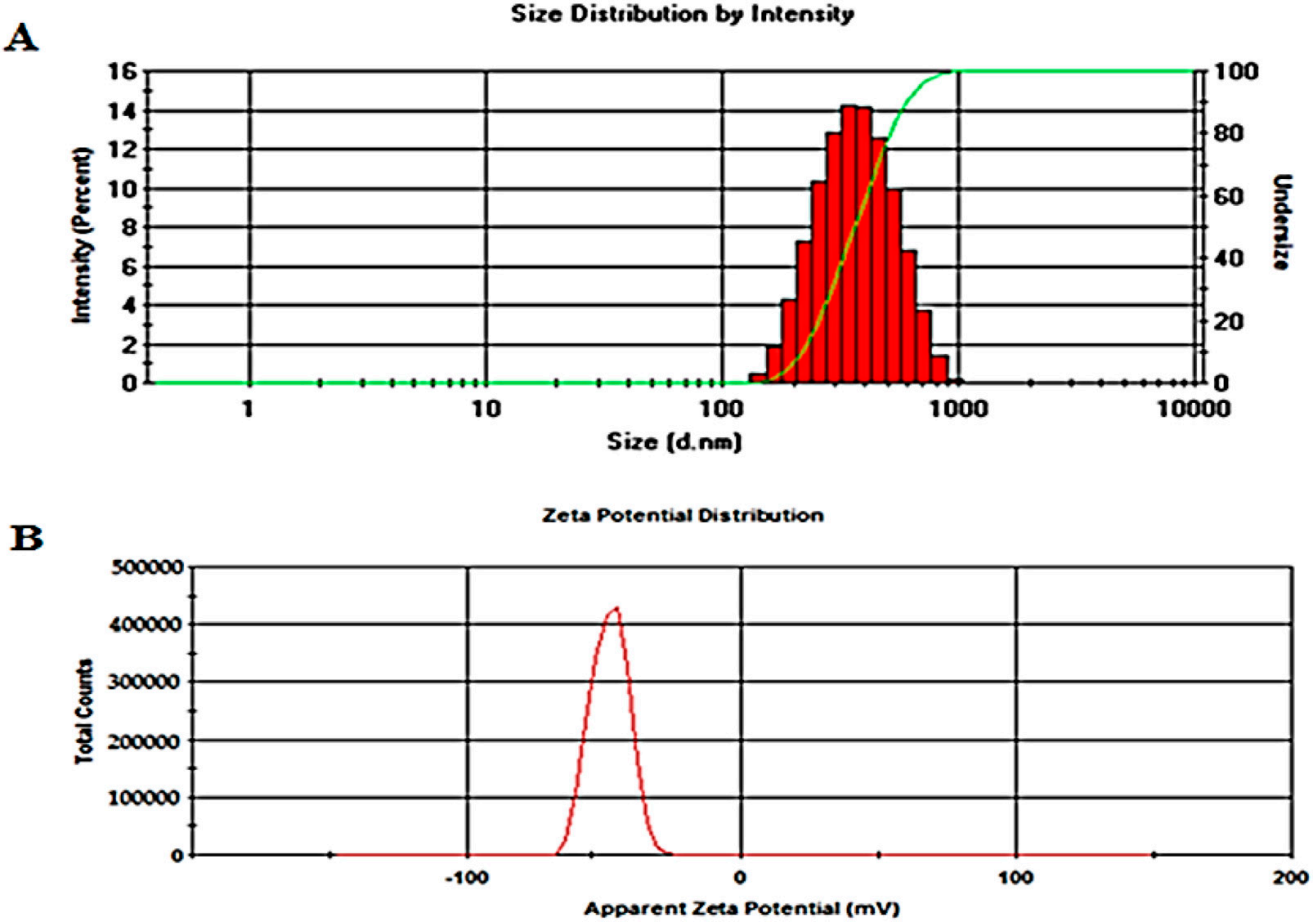
Vesicle size **(A)** and zeta potential **(B)** of the ZLP spanlastic optimized formula.

The optimum values of different independent factors were defined, 1.58% *w/v* of Span 60 and 1.0% *w/v* of Tween 80, using the design software for selecting the optimized formula based on minimizing vesicle size and maximizing EE % and % released over 24 h. The predicted values of responses were 303.92 nm, 67.96% and 75.62% for vesicle size, EE % and % released after 24 h, respectively. The observed results showed that the optimized formula had a size of 297.2 ± 8.17 nm (Figure 4), an EE % of 65.75 ± 3.28%, and a release rate of 76.44 ± 5.66%, indicating the applicability of the present model. Hence, the optimized formula was considered for further study [50].

The optimized spanlastic formulation has a high negative zeta potential value of −43.25 ± 3.6 mV, indicating good stability, Figure 4. The high zeta potential value is caused by the O-H of Span 60 and indicates good repulsion between charged vesicles, which can electrically stabilize the vesicles and prevent them from aggregating [51].

#### Morphology of the optimized ZLP-Loaded spanlastic formula

**FIGURE 5:**
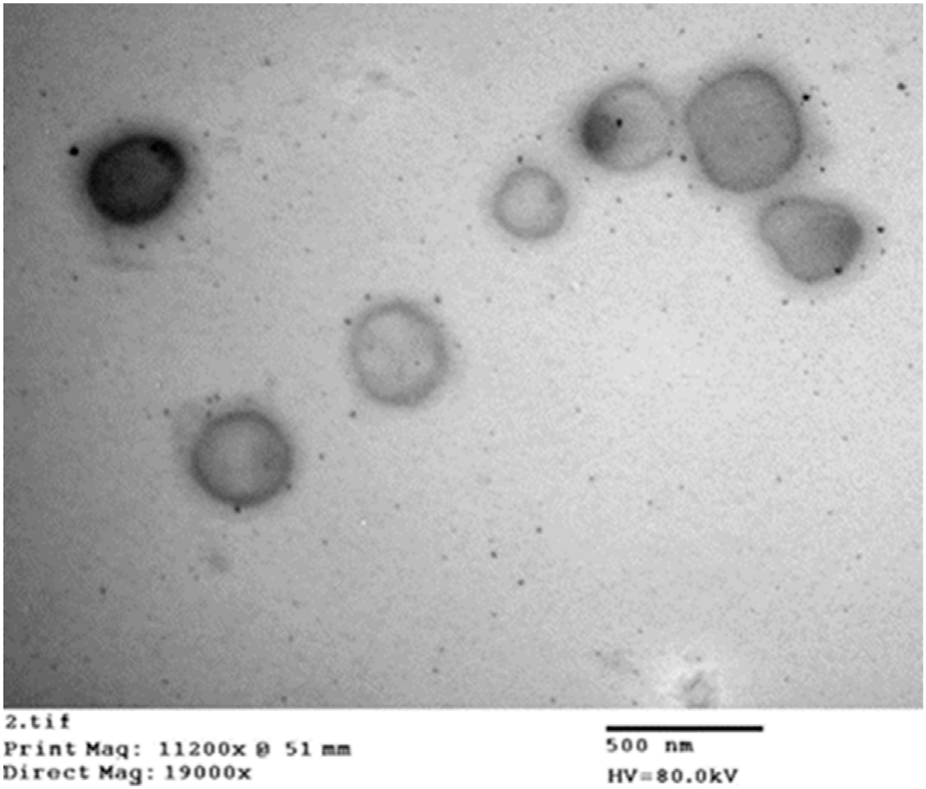
Transmission electron micrograph of the optimized ZLP spanlastic formula.

Figure 5 shows the TEM micrographs of the optimized ZLP-loaded spanlastics nanoformulation. The results revealed that the vesicles were spherical, without aggregation, and their sizes were in the nanometer range, which was consistent with the results reported by Zetasizer.

#### FT-IR spectroscopy

**FIGURE 6:**
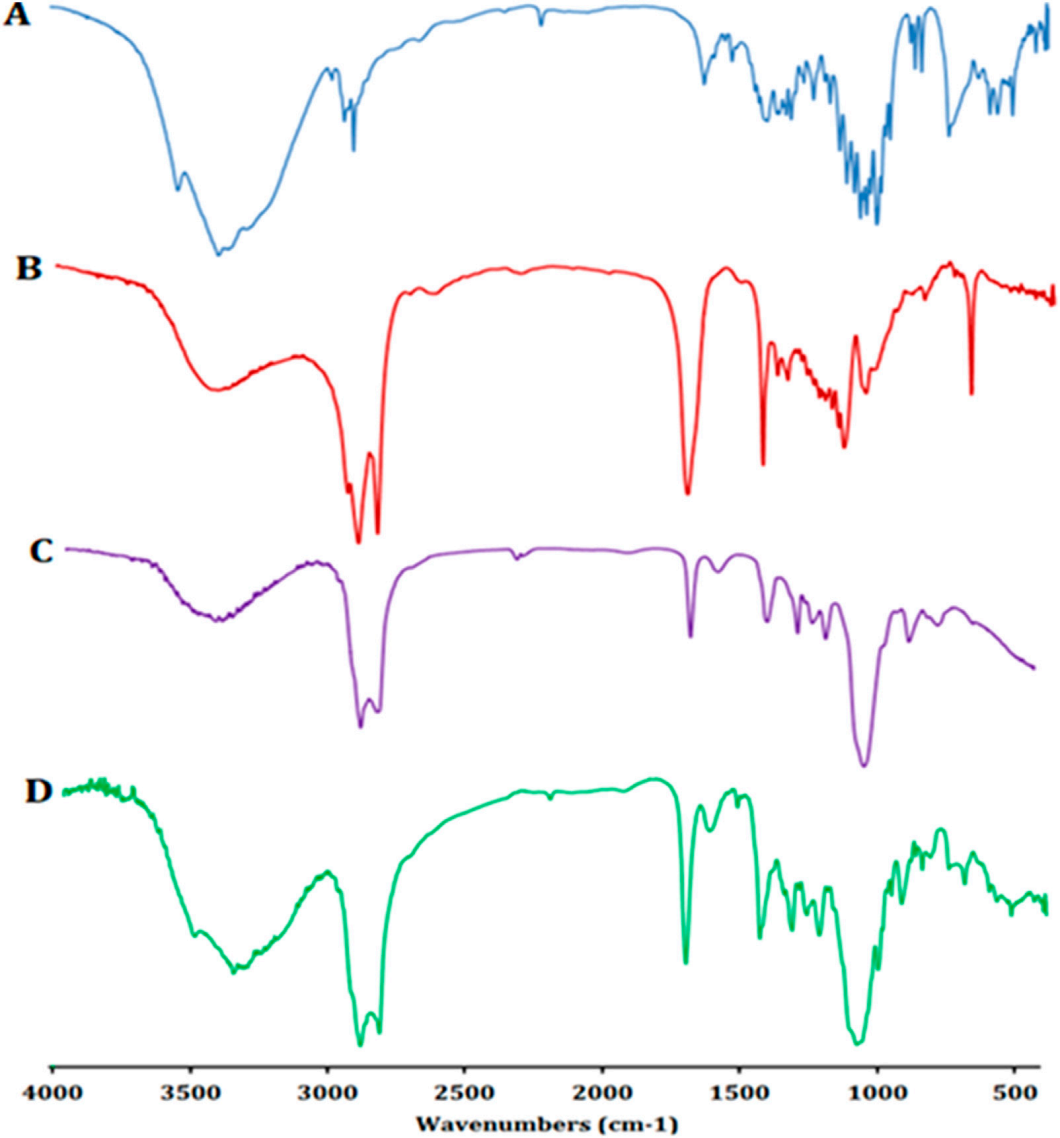
FT-IR spectra of pure ZLP **(A)**, Span 60 **(B)**, Tween 80 **(C)**, and ZLP spanlastic optimized formula **(D)**.

The FT-IR spectra of pure ZLP, surfactant (Span 60), EA (Tween 80), and the optimized ZLP spanlastic formula are displayed in Figure 6. FT-IR spectrum of ZLP is differentiated by absorption bands at 3339.28 cm^−1^ (C–H aromatic), 2,933.49 cm^−1^ (C–H aliphatic), 2,232.72 cm^−1^ (C≡N), 1,651.86 cm^−1^ (amide of C=O), 1,576.82 cm^−1^ (C=C aromatic) and 1,220.90 cm^−1^ (C–N). The broad peaks at 3,424.16 cm^−1^ and 3,420.58 cm^−1^ are attributed to O-H stretching vibrations of Span 60 and Tween 80, respectively [52]. The characteristic peaks of pure ZLP did not disappear in the optimized spanlastics formulation, indicating that there was no interaction between ZLP and the excipients used.

### Evaluation of ZLP- spanlastic transdermal patches

#### Physical appearance, thickness, pH, weight, and drug content uniformity

The prepared ZLP-SP formulations were found to be smooth, white, homogeneous, and flexible in their physical appearance. The thickness, pH, weight, and drug content of all ZLP-SP formulations were uniform with low SD values, as shown in Table 7. The *thickness* of the ZLP-SP formulations ranged from 0.131 ± 0.01 to 0.138 ± 0.02 mm. The surface pH values of the ZLP-SP formulations were within the acceptable range (4.7 ± 0.32 to 5.8 ± 0.25), indicating that the ZLP-SP formulations were suitable for use and did not cause skin irritation [53]. The *weights* of the prepared ZLP-SP formulations varied from 118.8 ± 1.14 to 130.7 ± 1.64 mg, indicating that an increase in HPMC K100M concentration resulted in an increase in the ZLP-SP formulation weight. The *drug content* was found to be ranging between 95.3 ± 2.1 to 96.1 ± 1.5 percent.

**TABLE 7:** Physicochemical evaluation of ZLP- spanlastic transdermal patches.

Formulae code	Thickness (mm)	pH	Weight (mg)	Drug content (%)	Elongation (%)	Moisture content (%)	Moisture uptake (%)
ZLP-SP1	0.131 ± 0.010	4.7 ± 0.32	118.8 ± 1.14	95.6 ± 2.5	35.2 ± 3.6	4.18 ± 0.416	5.97 ± 0.152
ZLP-SP2	0.134 ± 0.004	5.4 ± 0.41	125.2 ± 1.02	96.1 ± 1.5	33.5 ± 2.0	5.34 ± 0.371	6.93 ± 0.108
ZLP-SP3	0.138 ± 0.020	5.8 ± 0.25	130.7 ± 1.64	95.3 ± 2.1	29.3 ± 4.3	6.57 ± 0.562	7.15 ± 0.256

#### Elongation percent (E %)

Elongation testing can indicate the elasticity of the ZLP-SP formulations; meaning that a lower E % value would render the transdermal patch insufficiently resistant to breaking, resulting in a developed transdermal patch that tears easily. However, the increased E % values allow the polymer patch to withstand stretching. The E % of the prepared ZLP-SP formulations ranged from 29.3 ± 4.3 to 35.2 ± 3.6, Table 7, indicating that the transdermal patches had good structural integrity and could resist the stress applied during the manufacturing process. It was observed that there was an inverse relationship between polymer concentration and E %, so ZLP-SP3, which had the highest HPMC K100M concentration, had the lowest E %. Similar findings were reported by Shailesh T. et al., who developed transdermal patches of Repaglinide with comparable mechanical properties [54].

#### Moisture content (%) and moisture uptake (%)

The results obtained are depicted in Table 7. Moisture content percentage and moisture uptake percentage investigations indicated that there is a direct proportionality between the concentration of HPMC K100M (hydrophilic polymer) and percentage of moisture content and uptake. As the concentration of HPMC K100M increased, percentage of moisture content (4.18 ± 0.416% - 6.57 ± 0.562%) and moisture uptake (5.97 ± 0.152% - 7.15 ± 0.256%) of the investigated ZLP-SP formulations were increased. The low moisture content in the fabricated ZLP-SP formulations contributed to their stability while preventing the formation of completely dried and brittle patches [55].

### *Ex-vivo* permeation study

**FIGURE 7:**
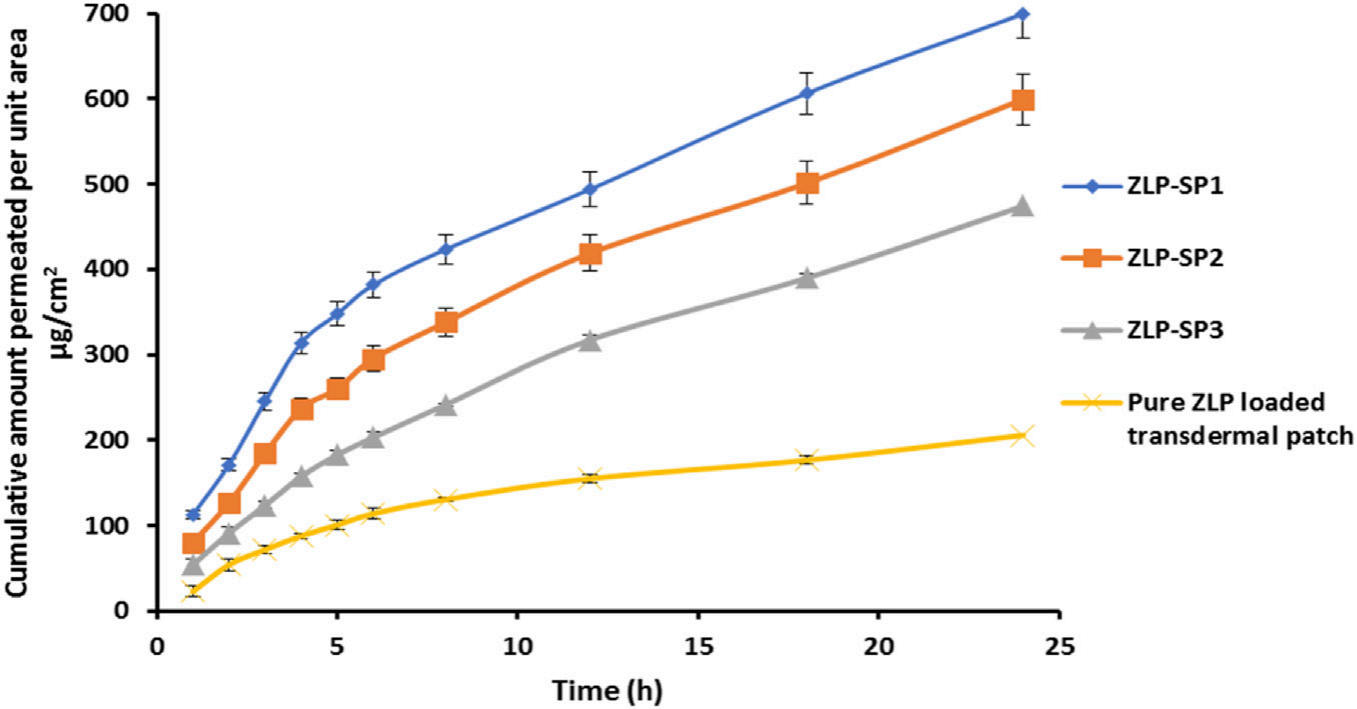
*In-vitro* permeation of ZLP from transdermal patches for 24 h.

Figure 7 illustrates the permeation pattern of the ZLP from the spanlastic-loaded transdermal patches in comparison with control transdermal patch (drug-loaded patch without spanlastics). The results obtained are represented in Table 8. It is obvious that the flux values of all spanlastic transdermal patches at all-time intervals are significantly higher than that of control transdermal patch. ZLP-SP1 showed higher permeation through the rabbit skin reaching 699.56 ± 6.54 μg/cm^2^ after 24 h. This may be attributed to the encapsulation of ZLP in flexible and elastic nano vesicles, which enhances the ZLP permeability and leads to a net increase in ZLP flux [14]. In line with previous reports, spanlastic formulations significantly enhanced *ex-vivo* skin permeation of drugs compared to free drug dispersions. For example, Tacrolimus-loaded spanlastics permeated rat skin more efficiently than the corresponding drug suspension [13]. Similarly, a spanlastic gel of Fenoprofen Calcium showed higher skin permeation than a conventional gel [11]. Furthermore, it was found that HPMC K100M concentration had a significant effect on the permeation results. As the concentration of HPMC K100M in the transdermal patches increases, the resistance of the matrix system to ZLP release increases, resulting in a decrease in the amount of drug permeated from the transdermal patches [56].

**TABLE 8:** Drug permeation parameters from spanlastic and control patches.

F. code	Flux (µg/cm^2^ h)	Apparent permeability (cm/h)	Enhancement ratio
ZLP-SP1	17.70 ± 0.24	0.00354	3.62
ZLP-SP2	16.56 ± 0.35	0.00331	3.34
ZLP-SP3	14.78 ± 0.37	0.00295	3.02
Pure ZLP-loaded transdermal patch	4.89 ± 0.11	0.00097	-

### Skin irritation evaluation

**FIGURE 8:**
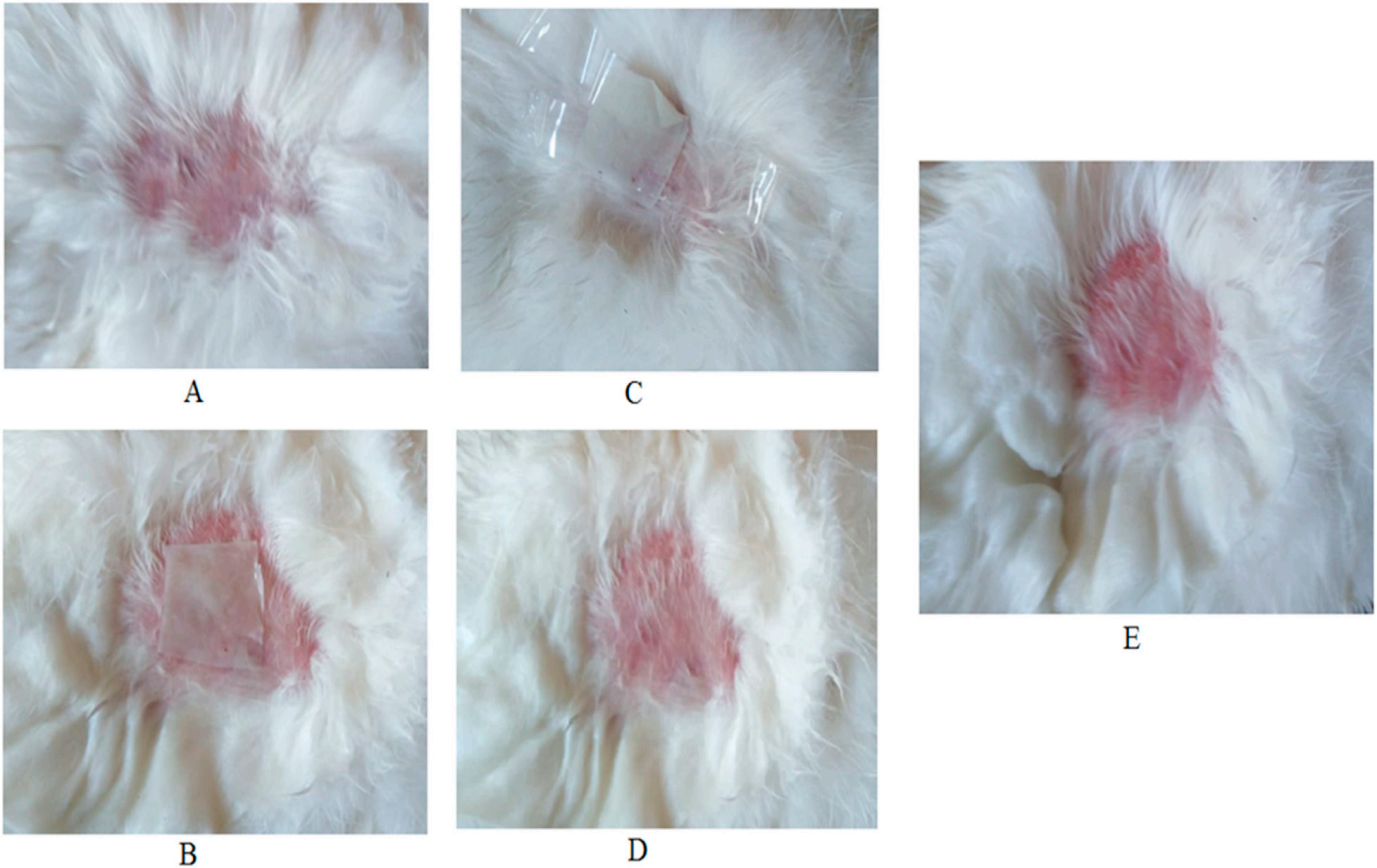
Photographs of rabbit skin irritation test: **(A)** Before application of transdermal patch, **(B)** application of placebo transdermal patch, **(C)** Application of ZLP-SP1, **(D)** After removal of ZLP-SP1, **(E)** After administration of formaldehyde (1% *v/v*).

The results of skin irritation tests of the ZLP-SP formulation (ZLP-SP1) in comparison with formaldehyde (1% *v/v*) showed that the erythema caused by the investigated ZLP-SP1 formulation was negligible, but that caused by formaldehyde was severe, group (B) (Figure 8). Formaldehyde causes high levels of irritation manifested by severe inflammation, epidermal discontinuity and skin ulcers. No irritation was observed in the animals of groups (A) and (C) that received placebo and ZLP-SP1, respectively. This indicates that the developed transdermal patch has non-irritating properties. This is supported by the findings of Adin et al., who found that topical application of baicalin-loaded transethosomal gel did not induce any skin irritation in animal models [57].

### Pharmacokinetic assessment

**FIGURE 9:**
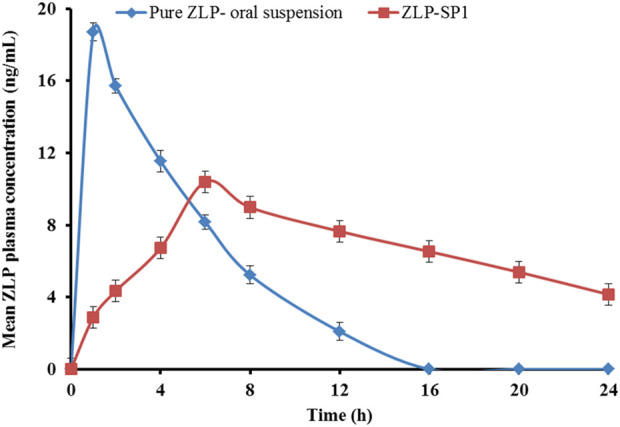
Mean plasma concentration-time profiles of ZLP after administration of pure ZLP oral suspension and the selected ZLP-SP1 formulation to rabbits. Values are mean ± SD for (n = 5).

Figure 9 shows the mean plasma concentrations of ZLP over time after oral administration of the pure ZLP suspension and transdermal application of the selected formulation (ZLP-SP1). The pharmacokinetic parameters studied are listed in Table 9.

**TABLE 9:** Pharmacokinetic parameters of ZLP different pharmaceutical formulations.

Pharmacokinetic parameters	ZLP formulation	
	Pure ZLP oral suspension	ZLP-SP1
C_max_ (ng/mL)	18.71 ± 2.16	10.4 ± 1.25
t_max_ (h)	1.0 ± 0.02	6.08 ± 0.378
K_el_ (h^-1^)	0.14035 ± 0.032	0.0473 ± 0.041
t_0.5_ (h)	4.937 ± 0.341	14.639 ± 0.226
AUC_0-24_ (ng × h/mL)	105.765 ± 6.53	283.55 ± 4.69
${\text{AUC}}_{0-\infty }$ (ng × h/mL)	753.58 ± 35.53	6,082.45 ± 57.65
Relative bioavailability (%)	-	2.681

There were significant differences in the pharmacokinetic parameters between the oral suspension and the ZLP-SP1, *P* < 0.05. It was observed that the maximum concentrations reached were 18.71 ± 2.16 and 10.4 ± 1.25 ng/mL at 1.0 ± 0.02 and 6.08 ± 0.378 h for the pure ZLP oral suspension and ZLP-SP1, respectively. This behavior might be due to the rapid absorption of ZLP via the oral route and the slow and sustained drug delivery via the transdermal route [13]^.^ In addition, the mean ${\text{AUC}}_{0-\infty }$ value, which reflects the extent of drug absorption, was significantly greater for ZLP-SP1 (6,082.45 ± 57.65 ng × h/mL) compared to the pure ZLP oral suspension (753.58 ± 35.53 ng × h/mL). This could be related to the avoidance of the first-pass effect associated with oral administration and the penetration-enhancing properties of the formulated ultra-deformable nanocarriers by overcoming the barrier function of the stratum corneum [58]. Also, the t_1/2_ was longer for ZLP-SP1 due to the slower elimination of ZLP from the body, which may be caused by the longer duration of absorption and continuous replenishment of ZLP in the systemic circulation from ZLP-SP1 over the period of application [59]. Furthermore, the calculation of relative bioavailability has shown that the bioavailability of ZLP-SP1 is approximately 2.681% higher than that of pure ZLP oral suspension. This is in agreement with the findings reported by Nermeen et al., who developed spanlastic gel formulations for the transdermal delivery of irbesartan, another BCS Class II drug. Their pharmacokinetic study demonstrated bioavailability enhancements of 1.72-fold and 2.53-fold compared to an oral suspension and a control gel, respectively [60].

### Stability study

The selected ZLP-SP1 formula was tested at 40 ± 0.5 °C and 75 ± 5% RH for 3 months to examine physicochemical parameters such as thickness, E %, drug content, weight, and moisture content and compared with the characteristics of freshly prepared formula. The results after the stability period indicated that the fabricated patches were stable under accelerated storage conditions, as shown in Table 10.

**TABLE 10:** Stability study of ZLP spanlastic transdermal patches (ZLP-SP1).

Parameter	Results at the end of study time (3 months)
Thickness (mm)	0.132 ± 0.01
Elongation (%)	34.1 ± 2.4
Drug content (%)	95.3 ± 1.3
Weight (mg)	124.2 ± 1.7
Moisture content (%)	3.56 ± 0.463

## Conclusion

ZLP-loaded spanlastic nanovesicles were successfully prepared using thin film hydration method. The formulae exhibited reasonable values of EE%, vesicle size, and zeta potential, and the drug amount released at 24 h. The spanlastic formulations were optimized using a 3^2^ factorial design. The optimal formulation contained Span 60 (1.58% *w/v*) and Tween 80 (1.0% *w/v*) exhibited adequate EE % (65.75 ± 3.28%), ViS (297.2 ± 8.17 nm), and sustained-release property over 24 h (76.44 ± 5.66%). The TEM image of the optimized spanlastic formula confirmed the spherical morphology of vesicles and that their size was comparable to that obtained by Zetasizer. The optimized spanlastic formula has been incorporated into transdermal patches. Transdermal patches of ZLP have been successfully formulated by solvent casting method. Evaluation of the fabricated patches in terms of physical appearance, uniformity of thickness, surface pH, weight uniformity, content uniformity, E %, and *ex-vivo* permeability study was performed. The obtained results suggest that the method utilized for patches preparation was reproducible and ensured excellent quality and uniformity in transdermal patches properties with negligible variability. The results after the stability period indicated that the fabricated patches were stable under accelerated storage conditions. Also, the developed transdermal patches have non-irritating properties. Further, the obtained results on *in-vivo* pharmacokinetics studies showed that the t_1/2_ was longer for transdermal patches due to the slower elimination of ZLP from the body, which may be caused by the longer duration of absorption and extended availability of ZLP in the systemic circulation from the patch over the period of application with a relative bioavailability approximately 2.681% higher than that of oral suspension. The obtained results revealed that the transdermal delivery of ZLP could be offered advantages in terms of reduced dosing frequency, improved patient compliance, non-invasive characteristics, improved bioavailability, and easy termination of therapy. Thus, it is concluded that the ZLP-spanlastic loaded transdermal patches could be a promising drug delivery system for ZLP for the management of insomnia.

## Data Availability

The original contributions presented in the study are included in the article/supplementary material, further inquiries can be directed to the corresponding author.
